# Capacity assessment and spatial analysis of cervical cancer services in The Gambia

**DOI:** 10.1186/s12905-023-02802-5

**Published:** 2023-12-09

**Authors:** Meghan E. Mali, Ousman Sanyang, Katherine L. Harris, Justin Sorensen, Mustapha Bittaye, Jonathan Nellermoe, Raymond R. Price, Edward K. Sutherland

**Affiliations:** 1 Center for Global Surgery, University of Utah School of Medicine, Salt Lake City, UT USA; 2 Department of Surgery, Edward Francis Small Teaching Hospital, Banjul, The Gambia; 3 Division of Gynecologic Oncology, Department of Obstetrics and Gynecology, Huntsman Cancer Institute, University of Utah, Salt Lake City, UT USA; 4 J. Willard Marriott Library, University of Utah, Salt Lake City, UT USA; 5 Ministry of Health and Social Welfare, Banjul, The Gambia; 6 Ensign Global College, Kpong, Eastern Region Ghana

**Keywords:** Cervical cancer, Access to care, Global oncology, Sub-Saharan Africa, The Gambia

## Abstract

**Background:**

Cervical cancer is the most common cancer and the leading cause of cancer-related death in Gambian women. The Gambian Ministry of Health is striving to improve access to screening, diagnostic, and treatment services for cervical cancer, but comprehensive data on currently available services is limited making it challenging to appropriately prioritize the ideal next steps for expanding care. This study aims to describe the current services available for the prevention, screening, and treatment of cervical cancer in The Gambia and provide suggestions for expanding geographic access to care.

**Methods:**

A survey aimed at assessing the availability of key cervical cancer-related services was developed and then administered in person by research assistants to all secondary and tertiary health facilities (HFs) in The Gambia. ArcGIS Pro Software and 2020 LandScan population density raster were used to visualize and quantify geographic access to care. Survey results were compared with published targets outlined by the Gambian Ministry of Health in the “Strategic Plan for the Prevention and Control of Cervical Cancer in The Gambia: 2016–2020.”

**Results:**

One hundred and two HFs were surveyed including 12 hospitals, 3 major health centers, 56 minor health centers, and 31 medical centers/clinics. Seventy-eight of these HFs provided some form of cervical cancer-related service. HPV vaccination was available in all health regions. Two-thirds of the population lived within 10 km of a HF that offered screening for cervical cancer and half lived within 10 km of a HF that offered treatment for precancerous lesions. Ten HFs offered hysterectomy, but nine were located in the same region. Two HFs offered limited chemotherapy. Radiotherapy was not available. If all major health centers and hospitals started offering visual inspection with acetic acid and cryotherapy, 86.1% of the population would live within 25 km of a HF with both services.

**Conclusions:**

Geographic access to cervical cancer screening, and precancer treatment is relatively widespread across The Gambia, but targeted expansion in line with the country’s “Strategic Plan” would improve access for central and eastern Gambia. The availability of treatment services for invasive cancer is limited, and establishing radiotherapy in the country should continue to be prioritized.

**Supplementary Information:**

The online version contains supplementary material available at 10.1186/s12905-023-02802-5.

## Background

Cervical cancer disproportionately affects low-resource countries. Incidence and mortality rates are estimated to be two to four times greater than in higher-resourced countries [1]. In The Gambia, cervical cancer is the most common cancer and leading cause of cancer-related death in women [2]. In addition, trends across SSA countries suggest an increasing incidence of the disease [3].

Rapid reduction in cervical cancer incidence and mortality over the last few decades in high-resourced countries is attributed partly to population-based cytology screening [4, 5]. Vaccination against human papillomavirus (HPV) is also considered central to cervical cancer elimination [6], although data on the impact of vaccination is just emerging due to the latency period for invasive disease [7]. Significant infrastructure and training investments are needed for successful vaccination and screening programs. Treatments must be developed in tandem to ensure that women diagnosed with precancerous lesions or invasive cancer can access care. This requires financial and human resources, planning, and governmental commitment [8, 9].

The Gambia is the smallest country on the African mainland but is also one of the most densely populated with over 2 million residents [10]. It is classified by the United Nations as a “least developed country” [11]. Approximately two-thirds of the population live in urban areas with 41% of the population living in the geographically small region Western Region 1 (WR1), where the capital city of Banjul is located [10]. Most governmental functions, secondary schools, universities, commercial activities, and tourism are located in the urban and semi-urban regions of WR1 and Western Region 2 (WR2). The remainder of the country is rural, and farming is a major source of revenue for these areas. The healthcare system includes both public and private health facilities. Within the public system, hospitals are expected to provide the majority of cervical cancer care. Major health centers may offer some care for cervical cancer, but less consistently than hospitals, and minor health centers are typically only expected to offer screening. Within the private system, medical centers/clinics typically offer services similar to or more comprehensive than general hospitals.

The Gambian government is committed to improving screening and care through the “Strategic Plan for the Prevention and Control of Cervical Cancer in The Gambia: 2016–2020” (Strategic Plan) [12]. Goals included ensuring nationwide access to HPV vaccination, improved access to screening with visual inspection with acetic acid (VIA), expansion of in-country pathology, and establishing the first center for radiotherapy in the country [12]. This paper presents the results of a comprehensive nationwide assessment of cervical cancer services in The Gambia performed in 2020 and assesses access to care based on the benchmarks described in the Strategic Plan. We highlight recent attainments and suggest additional ways to efficiently expand services.

## Methods

### Study design

A cross-sectional, health facility-based survey was administered at all publicly owned secondary and tertiary health facilities (HFs) and privately owned medical centers and clinics in The Gambia from March 9 to April 9, 2020. Tertiary HFs include the teaching hospital and general public hospitals. Secondary HFs provide “Basic Health Services” and include major and minor health centers. Primary HFs, including Village Health Services, were not surveyed [13]. A list of all secondary and tertiary HFs and all private medical centers/clinics was obtained from regional health directorates.

### Survey design and administration

The survey aimed to define healthcare personnel and services available at HFs in The Gambia to screen, diagnose, and treat breast and cervical cancer. Results of the breast cancer portion of the survey including details regarding survey development, design, administration, and training of research assistants (RAs), are published by Sanyang, et. al. [14]. In brief, the survey was modeled upon two existing surveys: The World Health Organization’s (WHO) “Tool for Situational Analysis to Assess Emergency and Essential Surgical Care”, [15] and Surgeons OverSeas’s “Personnel, Infrastructure, Procedures, Equipment, and Supplies Tool” [16]. The WHO’s first edition guidelines for the screening and treatment of pre-invasive cervical lesions, WHO’s guidance for the management of invasive cervical cancer, and the National Comprehensive Cancer Network’s Harmonized Guidelines for the treatment of cervical cancer in SSA were used to determine what key services to inquire about in the survey [17–19]. Specialists in cervical cancer in SSA and the United States reviewed the survey and provided feedback.

Seven RAs in total were recruited for the study, one familiar with each of the seven regions. They participated in a formal two-day training course demonstrating step-by-step administration of the survey. During this training, they participated in role-playing exercises to practice administering the questionnaire and were taught how to utilize their phones to obtain GPS coordinates for the HFs. Prior to returning to their regions to conduct the surveys, they administered the survey at one hospital under the supervision of the principal investigator.Availability of services was recorded based on average availability over the last year: 1) always available (available > 80% of the time), 2) not always available (available ≤ 80% of the time), and 3) not available. Respondents noted the number of healthcare personnel trained to perform specific procedures related to cervical cancer, even if the procedure was not offered at their place of employment. The full survey is available as Appendix 1.

Survey respondents were designated by each HF based on who was most knowledgeable on cervical cancer services at the facility. Trained RAs then conducted the survey in person and documented GPS coordinates.

### Data analysis and mapping of cervical cancer services

Descriptive data analysis was performed using Stata (version 16.1, 2019), and results are presented as frequency and proportions. Results were compared with the Strategic Plan and WHO’s most recent guidelines for cervical cancer screening and treatment [12, 18, 20].

Geographic information systems (GIS) technology was used to depict the location and distribution of services. Maps were created with ArcGIS Pro Software (Environmental Systems Research Institute 2021, Version 2.9.2), and proximity buffers extending in 5 km increments were generated. The 2020 LandScan population density raster from Oak Ridge National Laboratory (Oak Ridge, TN, USA) was utilized to estimate the landmass and population of each health region [21]. With a zonal statistics tool, the percentage of the population within various distances of services was estimated. Due to limited roads and barriers data available in The Gambia, distance from care was described as Euclidean distance rather than driving distance.

### Ethical approval

The Joint Committee of Research and Publication Committee University of The Gambia and Medical Research Council Ethics committees approved this research. Informed consent was provided by each HF and signed by the survey respondent. No patient-specific protected health information was accessed for this study.

## Results

### Health facilities

One hundred and two total HFs were identified by the regional health directorates that met criteria for the study as a secondary HF, tertiary HF, or private medical center/clinic. The response rate was 100% due to support from the Ministry of Health and regional health directorates. Of 102 HFs surveyed, minor health centers (56) and private medical centers/clinics (31) represented the majority of respondents. One teaching hospital, seven general hospitals, four district hospitals, and three major health centers also participated. Some form of cervical cancer care was offered at 78 HFs (76.5%). HPV vaccination was offered at 51 HFs (50.0%) as the only cervical cancer-related service, and 27 (26.5%) offered some form of screening, diagnostic, or treatment service other than just vaccination. Details of the 78 HFs that offered cervical cancer care and healthcare professionals reported in each region are summarized in Table 1.

**Table 1:** Summary of health facilities and healthcare professionals involved with cervical cancer care in the Gambia

**Health Region Name** Size of Health Region (km^2^)	**Population of Health Region (% of total population)**	**Number of Health Facilities offering cervical cancer services, by region**^a^	**Number of Healthcare Professionals potentially involved with cervical cancer care, by region**
**Western Region 1 (WR1)** 262 km^2^	883,593 (41.0%)	24 HFs - 1 teaching hospital - 2 general hospitals - 1 major H/C - 9 minor H/C - 11 medical centers/clinics	100 healthcare professionals - 25 Ob/Gyns - 3 Gyn/Oncs - 72 Midwives
**Western Region 2 (WR2)** 1556 km^2^	391,487 (18.2%)	9 HFs - 1 general hospital - 1 district hospital - 1 major H/C - 6 minor H/C	13 healthcare professionals - 0 Ob/Gyns - 0 Gyn/Oncs - 13 Midwives
**Upper River Region (URR)** 2079 km^2^	279,019 (12.9%)	11 HFs - 1 district hospital - 1 major H/C - 9 minor H/C	9 healthcare professionals - 0 Ob/Gyns - 0 Gyn/Oncs - 9 Midwives
**Central River Region (CRR)** 3028 km^2^	254,157 (11.8%)	11 HFs - 1 general hospital - 10 minor H/C	12 healthcare professionals - 1 Ob/Gyn - 1 Gyn/Onc - 10 Midwives
**North Bank West (NBW)** 1053 km^2^	127,685 (5.9%)	6 HFs - 1 district hospital - 5 minor H/C	6 healthcare professionals - 1 Ob/Gyn - 0 Gyn/Oncs - 5 midwives
**North Bank** **East (NBE)** 1210 km^2^	127,310 (5.9%)	8 HFs - 1 general hospital - 7 minor H/C	4 healthcare professionals - 2 Ob/Gyns - 1 Gyn/Onc - 1 Midwife
**Lower River Region (LRR)** 1495 km^2^	93,544 (4.3%)	9 HFs - 1 district hospital - 6 minor H/C - 2 medical centers/clinics	5 healthcare professionals - 0 Ob/Gyns - 0 Gyn/ Oncs - 5 Midwives
**Total**	**2,156,795**	**78 HFs** - 1 teaching hospital - 5 general hospitals - 4 district hospitals - 3 major H/C - 52 minor H/C - 13 medical centers/clinics	**149 healthcare professionals** - 29 Ob/Gyns - 5 Gyn/Oncs - 115 Midwives

^a^Offering cervical cancer services = any HF that offers at least ONE of the following: HPV vaccination, HPV screening, VIA, VILI, pap test, colposcopy, cervical biopsy, cryotherapy, cold knife cone biopsy, LEEP, surgery for cervical cancer, or chemotherapy for cervical cancer

### Pathology

The availability of pathology services was limited in The Gambia. In-house pathology was only available at the teaching hospital. Six HFs, all located in Western Region 1 (WR1), offered pathology as a send-out service to an external lab. Four sent samples to the teaching hospital, and two sent samples to a hospital in Senegal. Results typically took less than one month. One pathology specialist and one consultant employed at the teaching hospital were the only pathology personnel reported in The Gambia. 

### Imaging

Twelve HFs offered at least one imaging service. The teaching hospital had the most comprehensive services with ultrasound (US), x-ray, computed tomography (CT), and magnetic resonance imaging (MRI). Seven HFs had US and x-ray only. Two offered US, x-ray, and CT, one had x-ray only, and one had US only. The MRI scanner and all three CT machines were in WR1, however the MRI was not functional at the time of the survey. There were two regions (North Bank West and Upper River Region) with no imaging services. The HF that only offered US reported it was not always available. All other imaging services were reported as being available > 80% of the time. There were 19 radiology personnel in the country: 11 technicians, five specialists, and three consultants employed at eight total HFs (6 in WR1, 1 in Central River Region, and 1 in North Bank East).

### Prevention, screening, and treatment of precancerous lesions

HPV vaccination was available at 66 HFs (64.7%) and was the only cervical cancer service offered at 51 HFs (50.0%). The specific vaccine offered was not reported. Vaccination was available in every region.

**Fig. 1:**
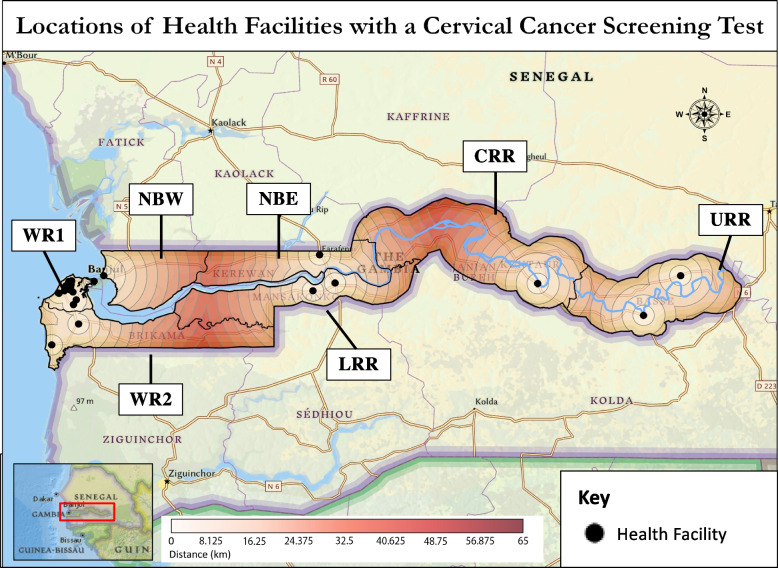
Locations of health facilities with a cervical cancer screening test. Screening tests include VIA, VILI, pap test, or HPV testing. Each circle depicts 5 km from the nearest health facility

Screening was performed at 24 HFs (23.5%) (Fig. 1). Methods included VIA, visual inspection with Lugol’s iodine (VILI), Papanicolaou smears (Pap smears), and HPV testing, including testing for specific high-risk subtypes. Of the 24 HFs that offered screening, 8 had VIA only, 8 had VIA and HPV testing, 1 had VIA and VILI, 1 had VIA, VILI, and HPV testing, 1 had VIA and Pap smear, 1 had Pap smear only, and 4 offered VIA, VILI, Pap smear, and HPV testing (3 included testing for high-risk subtypes). Clinician-collected HPV testing was the only type of HPV testing offered; self-swab was not available. These screening services were concentrated in WR1 (15/24 HFs, 62.5%), but every region had at least 1 HF with some form of screening. Colposcopy was available at 9 HFs, all located in WR1. All screening tests offered were reported to be always available.

Twenty-three HFs performed VIA, which was the most available screening test countrywide and was offered in each of the seven regions. Twenty-one of the HFs that offered VIA kept records of VIAs performed; 17 reported performing 1–25 tests per month, two reported 26–50, one reported 51–75, and one reported > 100. VIA testing was free at 17 HFs, cost 100–500 dalasi (about 2–10 USD) at four HFs, and > 500 dalasi (> 10 USD) at one HF, and one HF did not report cost data.

**Fig. 2:**
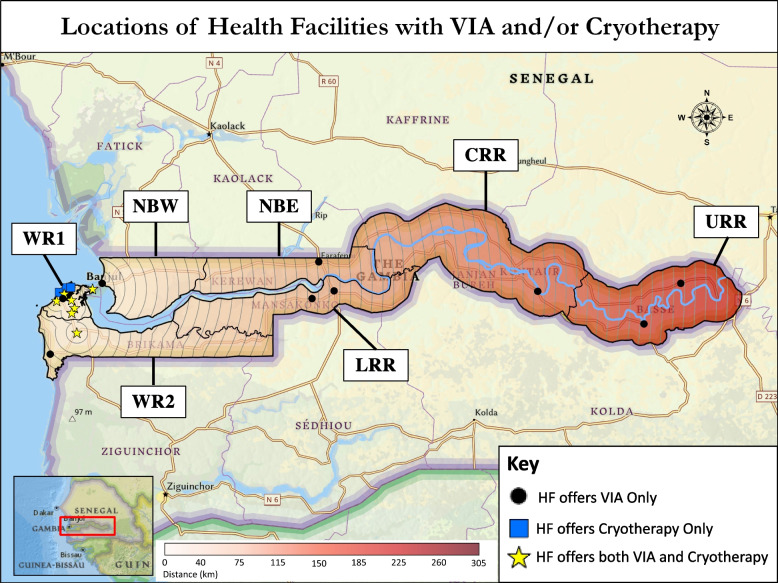
Locations of health facilities with VIA and/or cryotherapy. Each circle depicts 5 km from the nearest health facility that offers *both* VIA and cryotherapy

Fourteen HFs (13.7%) performed treatment of precancerous lesions of the cervix (one located in WR2, 13 (92.9%) in WR1). Treatments consisted of cryotherapy, cold knife cone biopsy (CKC), and loop electrosurgical excision procedure (LEEP). Of the 14 HFs that offered treatment, 11 had cryotherapy only, 1 had CKC only, 1 had both cryotherapy and CKC, and 1 had all three procedures. Of the treatment procedures offered, three HFs offering cryotherapy reported it was only available sometimes (< 80% of the time), but all other procedures were reported to be always available. Eleven HFs offered both a screening and a treatment method, ten of which offered both VIA and cryotherapy (Fig. 2).

### Treatment of invasive cancer

Surgery for cervical cancer was available at 10 HFs (nine located in WR1; all reported both simple and radical hysterectomy). Of the 10 HFs offering surgery, all employed at least one ob-gyn; only two employed a gynecologic oncologist. Two medical centers in WR1 reported offering chemotherapy for patients with cervical cancer. Both had cyclophosphamide, 5-FU, and methotrexate; one also had paclitaxel and cisplatin. No HFs in The Gambia offered radiotherapy and there were no radiation oncology specialists or consultants.

### Palliative care

Palliative care was offered at 13 HFs (12.7%), most located in WR1 (8). The Upper River Region had no palliative care services.

### Training of healthcare professionals

Forty-six HFs (45.1%) had personnel trained in at least one screening method, and 28 (27.4%) had personnel trained in at least one treatment method for the management of precancerous cervical lesions. For most HFs offering VIA, the procedure was performed by a midwife (18/23, 78.3%). Despite training of healthcare professionals in various screening and treatment modalities, many work at HFs that do not offer the service (Table 2). 173 total personnel were reported as trained in HPV vaccination administration.

**Table 2:** Training of healthcare professionals

Procedure	Total Number of Healthcare Professionals Trained in Procedure	Trained Professionals who work at HF that does NOT offer the service (n, % of trained professionals)
**HPV Screening**	46	11 (24.0%)
**VIA**	115	44 (38.3%)
**VILI**	31	3 (10.0%)
**Pap**	66	35 (53.0%)
**Colposcopy**	32	7 (21.9%)
**Cervical Biopsy**	33	7 (21.2%)
**Cryotherapy**	61	25 (41.0%)
**Cold Knife Cone**	15	2 13.3%)
**LEEP**	12	1 (8.3%)

### Population analysis

**Fig. 3:**
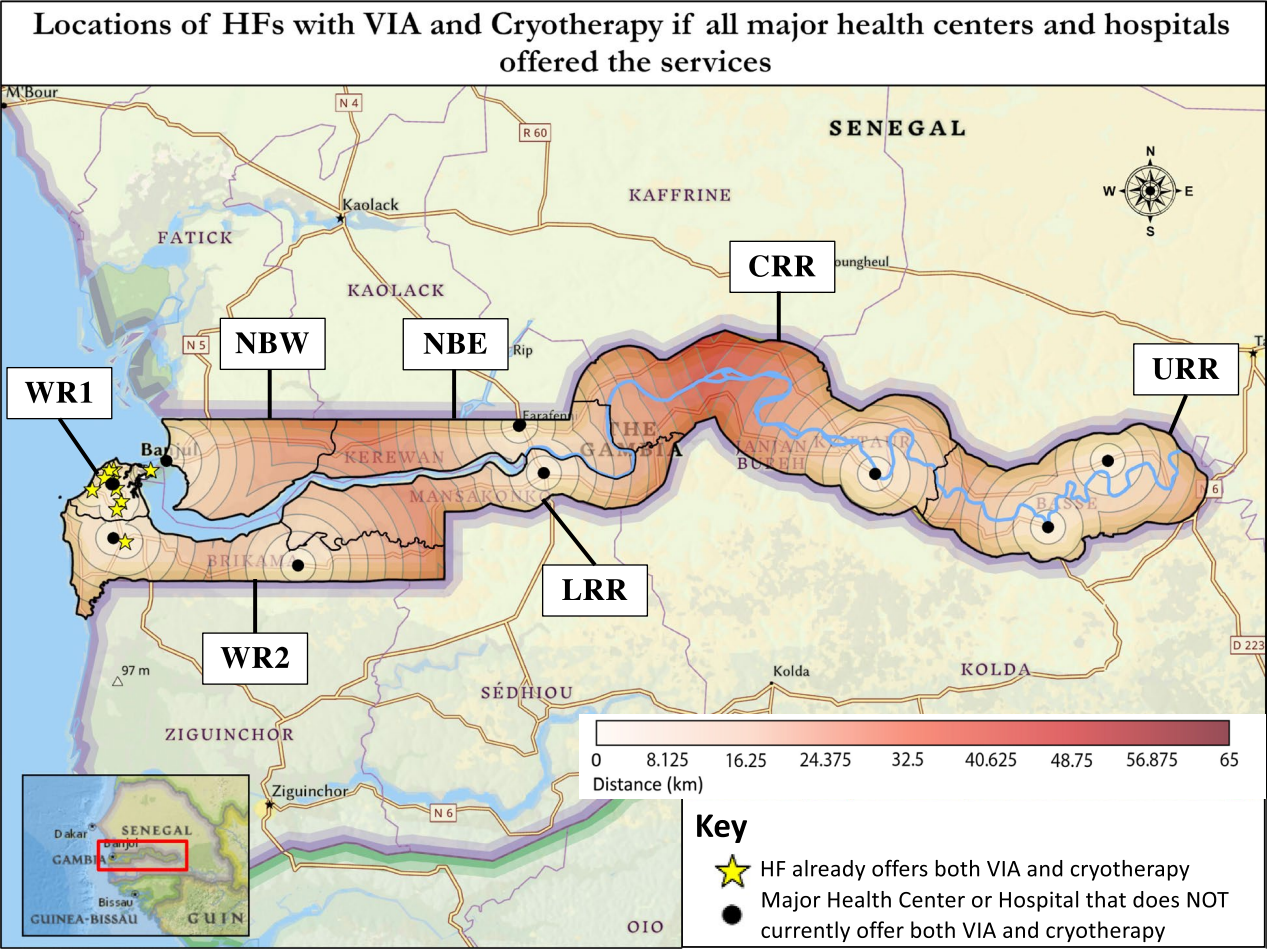
Visualization of *potential access* to VIA and cryotherapy if all major health centers and hospitals were equipped to offer *both* VIA and cryotherapy. Each circle depicts 5 km from the nearest health facility that would offer *both* VIA and cryotherapy in this potential scenario

Results of the population analysis of geographic access to care are detailed in Table 3. Nearly two-thirds of the population lived within 10 km of a HF that offered a screening test for cervical cancer. About half lived within 10 km of a HF with both VIA and cryotherapy, but access for the remaining population was more remote with a third > 75 km from a HF with both services. Surgical care within 10 km was more limited, but the proportion of those having to travel > 75 km for surgery was similar to that for a HF with both VIA and cryotherapy. A theoretical analysis was then performed to demonstrate access if all major health centers and hospitals started offering VIA and cryotherapy. This was chosen as a theoretical analysis because it is a stated goal in the Strategic Plan. In this scenario, 86.1% of the population would live within 25 km of a HF with both services and the entire population within 75 km (Fig. 3).

**Table 3:** Population analysis of geographic access to care

Service	Number of Health Facilities with Service	% of population living within specified Euclidean distance				
		0–10 km	10–25 km	25–50 km	50–75 km	> 75 km
Surgery for Cervical Cancer	10	38.5%	15.8%	7.3%	3.4%	35.0%
Any Screening Test^a^	24	65.6%	18.1%	14.4%	1.9%	–
VIA & Cryotherapy at the same HF^b^	10	52.0%	7.5%	3.7%	3.6%	33.2%
**Potential** access if VIA & Cryotherapy was offered at **ALL **Major Health Centers and Hospitals^c^	21	64.8%	21.3%	12.8%	1.1%	–

^a^Correlates with Fig. 1; screening tests include VIA, VILI, pap test, or HPV testing

^b^Correlates with Fig. 2

^c^Correlates with Fig. 3; represents a theoretical scenario, not current access

## Discussion

The Strategic Plan provides detailed goals aimed at mitigating the morbidity and mortality caused by cervical cancer in The Gambia [12]. Our survey evaluated the availability of key services for cervical cancer prevention, screening, and treatment. These results can be viewed alongside the Strategic Plan’s stated goals to evaluate progress and frame next steps to improve access.

For the primary prevention of cervical cancer, the Gambia succeeded in its goal to have vaccination available in every region [12]. In addition, an HPV vaccination program with the quadrivalent vaccine was introduced in The Gambia in 2019. This program is primarily delivered in schools and is targeted at girls aged 9 – 13 years old. By 2022, nearly three-quarters of females received at least one dose of the vaccination by age 15. Completion of the series has been much lower with only 28% of females receiving the second dose by age 15, but the most recent WHO recommendations offer a single-dose schedule as an acceptable alternative to the more typical two-dose regimen [22, 23]. With vaccination available in every region and an established school-based vaccination program, continuing to address misconceptions and concerns about the vaccination may be an important focus to further increase uptake of the vaccine [24].

For secondary prevention of cervical cancer through screening and treatment of pre-cancer lesions, the Strategic Plan aimed to have VIA and cryotherapy at all major health centers (MHCs) and hospitals in the country [12]. Availability of both services at the same HF allows for screening and treatment within a single visit, simplifying care for patients. Our survey found that 11 of the 15 (73.3%) MHCs and hospitals had VIA, and only 4 (26.7%) offered cryotherapy. Some minor health centers and medical centers/clinics also offered these services. Of the 10 HFs that offered both VIA and cryotherapy, nine were in WR1, and one was in WR2. If all MHCs and hospitals offered VIA and cryotherapy, HFs offering both would be available in every region, and the population living within 25 km of a HF with both services would increase from 59.5% to 86.1% (Table 3, Figs. 2 and 3).

Establishing VIA and cryotherapy across all MHCs and hospitals helps ensure adequately distributed geographic access. This will require HFs have the appropriate equipment and adequately trained staff. Many healthcare professionals trained in these procedures are working at HFs that did not offer the service (Table 2). This finding represents a unique opportunity to expand care by targeting HFs that already have trained professionals; however, all of these trained professionals are located in western Gambia, where adequate services exist. The Strategic Plan detailed goals related to the training of healthcare professionals to: 1) have at least 2 staff trained in screening and cryotherapy at 50% of all HFs and 2) train midwives and nurses in private hospitals and health centers on VIA [12]. To achieve the goal of having VIA and cryotherapy available at all MHCs and hospitals, we suggest focusing efforts on further training for healthcare professionals employed at MHCs and hospitals, particularly in the central and eastern health regions. Trained providers in the western part of the country could be deployed to train personnel in the central and eastern regions, although care must be taken to ensure skills remain proficient among those who are not regularly utilizing these in their daily clinical practice.

The WHO’s updated guidelines for screening and treatment of precancer lesions shifted the focus from VIA to HPV testing as the recommended initial screening test [20]. HPV testing was offered at 13 HFs in The Gambia, but was concentrated in the west and not available in every region. Given this re-prioritization by the WHO, the cost-effectiveness and feasibility of expanding HPV versus VIA testing is an additional consideration for health officials in The Gambia.

Similar to vaccination, screening availability does not ensure utilization. Current estimates show that only 11% of women have ever received cervical cancer screening in their lifetime [25]. Lack of knowledge about the disease, concerns regarding privacy, cultural and religious beliefs, and cost have all been described as barriers to utilizing cervical cancer screening services in SSA [26–28]. Self-collected HPV testing has the potential to improve screening uptake [29] and PCR-based tests have comparable sensitivity and specificity with clinician-collected samples [30], but these forms of testing were not available at any of the surveyed HFs. The feasibility of introducing self-collected tests should be evaluated as the country continues increasing screening.

Limited access to pathology services in the Gambia highlights the value of screening approaches not reliant upon cytologic technologies. The Strategic Plan goals included: 1) having functional pathology labs in at least 3 major referral hospitals, 2) hiring three additional pathologists, and 3) sending two Gambian doctors to be trained as pathologists. There was only one functional pathology laboratory in the country with one pathology specialist and one consultant. All HFs with established referral systems to external laboratories were located in WR1. Expanding cytology and histopathology in the country would be beneficial for the diagnosis and prognostication of numerous diseases in addition to cervical cancer, but the development of these services is time and resource-intensive [31]. Improving referral systems to external pathology laboratories for central and eastern Gambia will be important as additional laboratories are developed. Alternatively, if local labs have capability to process and create slides, infrastructure could be established to allow virtual interpretation of the slides by out-of-country pathologists until more in-country pathologists can be trained.

For the management of invasive cervical cancer, the Strategic Plan aimed to have three major referral hospitals “equipped” for radical hysterectomy [12]. Our survey identified only two of the three major referral hospitals offered simple and radical hysterectomy. The Strategic Plan called for an effort to start radiotherapy at the tertiary level and to have two Gambian doctors trained in radiotherapy. Unfortunately, radiotherapy is not available in The Gambia. The current practice is for patients to be referred to other countries, most often to neighboring Senegal, for most chemotherapy and all radiotherapy, but this is not a financially viable option for some patients. Further studies to detail referral practices, costs, and the frequency in which patients are able to complete non-surgical treatment in other countries would clarify current access to chemotherapy and radiation therapy for women with cervical cancer in The Gambia.

Lastly, the Strategic Plan calls for all hospitals to provide palliative care. Our survey found that only 5 of the 12 hospitals (41.7%) offered palliative care. Further evaluation of specific services available will clarify where advancements in care can be made.

### Limitations

Many factors impact both access and utilization of healthcare, with distance being only one element [26–28, 32]. Information about advocacy and awareness skills training for healthcare professionals was not elicited in this study but is important in encouraging utilization of available care. When considering geographic access, drive time is preferable, but limited roads and barriers data in The Gambia leads to largely inaccurate results. Euclidean distance has reasonable correlation with drive time but has reduced accuracy for estimating distance in areas with traffic congestion [33]. Our analysis likely overestimates access for those within the shortest straight-line distance of care, specifically the population living in WR1. Despite these limitations, our analysis including various distance ranges coupled with population density (Table 3) can be used by the Gambian Ministry of Health to make informed decisions about access in the country and how to best expand care.

## Conclusions

This study evaluated the screening, diagnostic, and treatment services available for cervical cancer in The Gambia. These results can guide the formulation of updated goals for The Gambia to maximize access for its population. Priorities highlighted focus on vaccine distribution, screening and treatment for precancer lesions at all MHCs and hospitals, improvements of pathology referral systems, and establishment of radiotherapy. Targeted incremental improvements in access to comprehensive care will support the Gambia’s national project of cervical cancer elimination.

### Supplementary Information

**Additional file 1. **

## Data Availability

Data including the facility-level results of the survey are maintained by the Gambian Ministry of Health. Reasonable requests for access to the data can be made to the corresponding author. This is in order to maintain the requested anonymity of participating health facilities. Population density and landmass datasets that were utilized for this project are publically available as the 2020 LandScan population density raster from Oak Ridge National Laboratory (Oak Ridge, TN, USA). This data can be downloaded for free by accessing https://landscan.ornl.gov or https://doi.org/10.48690/1523378. On this site, the user can click “Download” in the top right corner and select “LandScan Global” and the year 2020 to obtain the same data file we used for this study.

## Abbreviations

CKC: Cold knife cone biopsy
CT: Computed tomography
HF: Health facility
HPV: Human papillomavirus
LEEP: Loop electrosurgical excision procedure
MHC: Major health centers
MRI: Magnetic resonance imaging
Pap smear: Papanicolaou smear
SSA: Sub-Saharan Africa
RA: Research assistant
Strategic Plan: “Strategic Plan for the Prevention and Control of Cervical Cancer in The Gambia: 2016–2020”
US: Ultrasound
VIA: Visual inspection with acetic acid
VILI: Visual inspection with Lugol’s iodine
WHO: The World Health Organization
WR1: Western Region 1
WR2: Western Region 2

## References

[1] Arbyn M, Weiderpass E, Bruni L, de Sanjose S, Saraiya M, Ferlay J, Bray F (2020). Estimates of incidence and mortality of cervical cancer in 2018: a worldwide analysis. Lancet Glob Health.

[2] Observatory TGC (2020). GLOBOCAN 2020: estimated cancer incidence, mortality, and prevalence worldwide in 2020.

[3] Jedy-Agba E, Joko WY, Liu B, Buziba NG, Borok M, Korir A, Masamba L, Manraj SS, Finesse A, Wabinga H (2020). Trends in cervical cancer incidence in sub-Saharan Africa. Br J Cancer.

[4] Bray F, Loos AH, McCarron P, Weiderpass E, Arbyn M, Moller H, Hakama M, Parkin DM (2005). Trends in cervical squamous cell carcinoma incidence in 13 European countries: changing risk and the effects of screening. Cancer Epidemiol Biomarkers Prev.

[5] Bray F, Ferlay J, Soerjomataram I, Siegel RL, Torre LA, Jemal A (2018). Global cancer statistics 2018: GLOBOCAN estimates of incidence and mortality worldwide for 36 cancers in 185 countries. CA Cancer J Clin.

[6] Brisson M, Kim JJ, Canfell K, Drolet M, Gingras G, Burger EA, Martin D, Simms KT, Benard E, Boily MC (2020). Impact of HPV vaccination and cervical screening on cervical cancer elimination: a comparative modelling analysis in 78 low-income and lower-middle-income countries. Lancet.

[7] Oliveira CR, Niccolai LM (2021). Monitoring HPV vaccine impact on cervical disease: Status and future directions for the era of cervical cancer elimination. Prev Med.

[8] Sankaranarayanan R, Anorlu R, Sangwa-Lugoma G, Denny LA (2013). Infrastructure requirements for human papillomavirus vaccination and cervical cancer screening in sub-Saharan Africa. Vaccine.

[9] Randall TC, Ghebre R (2016). Challenges in prevention and care delivery for women with cervical cancer in Sub-Saharan Africa. Front Oncol.

[10] Andrew Clark ERAF, Harry A, Britannica TEOE (2023). Gailey: The Gambia. Britannica.

[11] Department of Economic and Social Affairs Committee for Development Policy. The least developed country category: 2021 Country snapshots. United Nations; 2021. https://www.un.org/development/desa/dpad/least-developed-country-category-gambia.html.

[12] The Gambia Ministry of Health and Social Welfare: strategic plan for the prevention and control of cervical cancer in The Gambia: 2016–2020. https://extranet.who.int/ncdccs/Data/GMB_B5_CSP%20of%20The%20Gambia-%20%202016%20%E2%80%93%202020%20Final.pdf.

[13] Comprehensive Multi-Year Plan for Immunizations (2017–2021), The Gambia. 2016. http://staging.nationalplanningcycles.org/sites/default/files/planning_cycle_repository/gambia/the_gambia_cmyp_2017-2021.pdf.

[14] Sanyang O, Lopez-Verdugo F, Mali M, Moustafa M, Nellermoe J, Sorensen J, Bittaye M, Njie R, Singhateh Y, Sambou NA (2021). Geospatial analysis and impact of targeted development of breast cancer care in The Gambia: a cross-sectional study. BMC Health Serv Res.

[15] Organization TWH (2009). Integrated Management for Emergency and Essential Surgical Care (IMEESC) toolkit. Tool for Situational Analysis to Assess Emergency and Essential Surgical Care.

[16] PIPES Surgical Assessment. http://www.adamkushnermd.com/files/PIPES_tool_103111.pdf.

[17] World Health Organization (2013). WHO guidelines for screening and treatment of precancerous lesions for cervical cancer prevention.

[18] World Health Organization (2014). Comprehensive cervical cancer control: a guide to essential practice.

[19] National Comprehensive Cancer Network (2019). NCCN harmonized guidelines for Sub-Saharan Africa: cervical cancer.

[20] World Health Organization (2021). WHO guideline for screening and treatment of cervical pre-cancer lesions for cervical cancer prevention.

[21] Rose A, McKee J, Sims K, Bright E, Reith A, Urban M (2021). LandScan Global 2020. Edited by Oak Ridge National Laboratory.

[22] Human Papillomavirus (HPV) vaccination coverage. https://immunizationdata.who.int/pages/coverage/hpv.html?CODE=GMB&ANTIGEN=15HPVC_F&YEAR=.

[23] WHO updates recommendations on HPV vaccination schedule. https://www.who.int/news/item/20-12-2022-WHO-updates-recommendations-on-HPV-vaccination-schedule.

[24] Wilson RJ, Leigh L, Bah H, Larson HJ, Clarke E (2023). HPV vaccination acceptance and perceptions related to fertility and population control in the Gambia: An anthropological analysis. Vaccine.

[25] ICO/IARC Information Centre on HPV and cancer: Gambia human papillomavirus and related cancers, fact sheet 2023. IHPV Information Centre; 2023. https://hpvcentre.net/statistics/reports/GMB_FS.pdf.

[26] Lim JN, Ojo AA: Barriers to utilisation of cervical cancer screening in Sub Sahara Africa: a systematic review. Eur J Cancer Care (Engl) 2017;26(1). 10.1111/ecc.12444 26853214

[27] Binka C, Nyarko SH, Awusabo-Asare K, Doku DT (2019). Barriers to the Uptake of Cervical Cancer Screening and Treatment among Rural Women in Ghana. Biomed Res Int.

[28] Isa Modibbo F, Dareng E, Bamisaye P, Jedy-Agba E, Adewole A, Oyeneyin L, Olaniyan O, Adebamowo C (2016). Qualitative study of barriers to cervical cancer screening among Nigerian women. BMJ Open.

[29] Gizaw M, Teka B, Ruddies F, Abebe T, Kaufmann AM, Worku A, Wienke A, Jemal A, Addissie A, Kantelhardt EJ (2019). Uptake of Cervical Cancer Screening in Ethiopia by Self-Sampling HPV DNA Compared to Visual Inspection with Acetic Acid: A Cluster Randomized Trial. Cancer Prev Res (Phila).

[30] Arbyn M, Verdoodt F, Snijders PJ, Verhoef VM, Suonio E, Dillner L, Minozzi S, Bellisario C, Banzi R, Zhao FH (2014). Accuracy of human papillomavirus testing on self-collected versus clinician-collected samples: a meta-analysis. Lancet Oncol.

[31] Stalsberg H, Adjei EK, Owusu-Afriyie O, Isaksen V (2017). Sustainable Development of Pathology in Sub-Saharan Africa: An Example From Ghana. Arch Pathol Lab Med.

[32] Regmi KRR, Gurch: Access to Healthcare: Issues of Measure and Method. Prim Health Care Open Access. 2013;3(1).

[33] Phibbs CS, Luft HS (1995). Correlation of travel time on roads versus straight line distance. Med Care Res Rev.

