# Supplementary material for: Capacity assessment and spatial analysis of cervical cancer services in The Gambia
Source: BMC Womens Health. 2023 Dec 9;23:660. doi: 10.1186/s12905-023-02802-5 (PMC10709932; doi:10.1186/s12905-023-02802-5)
Supplement: Supplementary file 1 — Additional file 1. [file 12905_2023_2802_MOESM1_ESM.pdf]

Appendix 1 – Survey

**Nationwide Assessment and Mapping of Breast and Cervical Cancer Services in The Gambia**<br>*(To be completed by Chief Medical Director , CEOs, Chairperson MAC, Officer in-charge or Clinical Coordinators)*	
Region:	
District:	
Date:	
Name of Respondent:	
Contact Telephone Number of Respondent:	
Title/Position:	
Name of Facility:	
Local Address:	
Digital Address (GPS*): (*Very Important)	
Telephone Number of facility(if any):	
Research assistant's name:	
Research assistant's contact number:	
Site visit/ in-person interview?	
Was any of the interview performed over the phone? **Y**=yes/**N**=No	

Healthcare facility type:<br>1. Teaching Hospital<br>2. General Hospital<br>3. District Hospital<br>4. Major Health Center<br>5. Minor Health Center<br>6. Medical Center/ Clinic	
Ownership of health care facility<br>1. Government<br>2. Quasi-Government<br>3. Private<br>4. Faith based (Mission)<br>5. Others; please specify	
Does your facility run breast clinic?<br>**Y**=Yes / **N**=No	
Does your facility attend to patients with breast cancer in other (general) clinics? **Y**=Yes / **N**=No	
Does your facility run cervical cancer clinic?<br>**Y**=Yes / **N**=No	
Does your facility attend to patients with cervical cancer in other (general) clinics? **Y**=Yes / **N**=No	

**Personnel**	**Number (How many)?**		
Trained general surgeon			
Physician assistant surgeon			
Trained Plastics/Reconstructive Surgeon			
Trained Obs/Gyn Surgeon			
Trained gynecologist-oncologist			
Radiologist	Technician	Specialist	Consultant
Pathologist	Lab technician	Specialist	Consultant
Oncologist	Specialist	Consultant	
Radiation oncologist	Specialist	Consultant	
Social worker/care coordinator			
Midwife			
Others (Specify)			

**Breast Cancer Screening and Imaging**			
**Does your center provide the following services:**	**Y=Yes / N=No**	**Always available (>80% of the time) Indicate with =1**	**Not always available Indicate with =0**
Clinical breast exam(CBE)			
Mammography			
If yes mammography:	complete mammography sub survey as well		
Ultrasonography(Ultrasound scan[USS])			
X Ray			
MRI			
CT-scan			
Genetic testing			

**Mammography Sub Survey**	
Does your facility keep records of mammograms performed? **Y**=Yes / **N**=No	
Number of mammograms per month: 1) 0-25<br>2) 26-50<br>3) 51-75<br>4) 75-100<br>5) >100	
Is the service provided free? **Y**=Yes / **N**=No	
If no, what is the cost to patient? 1) ≤1000 GMD<br>2) >1000-5000 GMD	

3 > 5000 GMD	
Is the cost covered by Insurance?<br>**Y**=Yes / **N**=No	
Type of insurance? 1 Private<br>2 Others; please comment	
Is the Insurance coverage 100%?<br>**Y**=Yes / **N**=No	
Does your facility conduct in-house review of mammogram?<br>**Y**=Yes / **N**=No	
Who interprets mammogram in-house? (List all applicable)<br>1 In house consultant radiologist<br>2 In house specialist radiologist<br>3 In house non radiologist (such as surgeon)<br>4 Others; please specify	
If external review:<br>1 Within Gambia?<br>2 Outside Gambia?	
Facility name and country (if outside Gambia)	
Time for results? 1) < 2 weeks<br>2) 2weeks - 1 month<br>3) > 1 month	

Cervical Cancer Screening		
**Does your facility have trained staff in the following Services**	**Y= Yes / N=No**	**Number of Trained Staff**
Pap’s Smear		
VIA		
VILI		
Colposcopy		
Cervical Biopsy		
LEEP		
Cold Knife Cone		
Cryotherapy		
HPV Screening		
HPV subtype 16/18 screening		
HPV Vaccination		
Others (specify eg Cinluma)		

Cervical Cancer Screening			
**Does your center provide the following services:**	**Y=Yes / N=No**	**Always available (>80% of the time) Indicate with =1**	**Not always available Indicate with =0**
HPV screening			
If yes, how is the HPV screening conducted?<br>1. Performed by provider<br>2. Patient self-swab			
Do you screen for the most common HPV subtypes isolated (e.g. 16, 18, etc.)			
Pap’s smear			
If yes to Pap’s smear **complete** Pap’s smear **sub survey** as well			

VIA (Visual Inspection with Acetic Acid)			
If yes to VIA **complete** VIA **sub survey** as well			
VILI (Visual Inspection with Lugol's Iodine)			
If yes to VILI **complete** VILI **sub survey** as well			

**Pap Smear sub survey**	
Does your facility keep records of Pap smears performed?<br>**Y=**Yes / **N=**No	
Number of Pap smears per month<br>1 0-25<br>2 26-50<br>3 51-75<br>4 75-100<br>5 >100	
Is the service provided free?<br>**Y**=Yes / **N**=No	
If no, what is the cost to patient?<br>1 ≤100 GMD<br>2 >100-500 GMD<br>3 > 500 GMD	
Is the cost covered by Insurance?<br>**Y**=Yes / **N**=No	

Type of insurance?<br>1 Private<br>2 Others; please comment	
Is Insurance coverage100%?<br>**Y**=Yes / **N**=No	
Who performs the Pap Smear?<br>1) Trained nurse<br>2) Midwife<br>3) Gynecologist<br>4) Others; please specify	

**VIA Sub Survey**	
Does your facility keep records of VIAs performed?<br>**Y**=Yes / **N**=No	
Number of VIAs performed per month?<br>1 0-25<br>2 26-50<br>3 51-75<br>4 75-100<br>5 >100	
Is the service provided free?<br>**Y**=Yes / **N**=No	
If no, what is the cost to patient?<br>1 100 GMD<br>2. >100-500 GMD<br>3. > 500 GMD	

Is the cost covered by Insurance?<br>**Y**=Yes / **N**=No	
Type of insurance?<br>1) Private?<br>2) Other; please indicate	
Is Insurance coverage100%?<br>**Y**=Yes / **N**=No	
Who performs the VIA?<br>1) Trained nurse<br>2) Midwife<br>3) Gynecologist<br>4) Others, please specify	

**VILI Sub Survey**	
Does your facility keep records of VILIs performed?<br>**Y**=Yes / **N**=No	
Number of VILIs performed per month?<br>1 0-25<br>2 26-50<br>3 51-75<br>4 75-100<br>5 >100	
Is the service provided free?<br>**Y**=Yes / **N**=No	

If no, cost to patient<br>1. 100 GMD<br>2. >100-500 GMD<br>3. > 500 GMD	
Is the cost covered by Insurance?<br>**Y**=Yes / **N**=No	
Type of insurance?<br>1. Private?<br>2. Others; please indicate	
Is Insurance coverage100%?<br>**Y**=Yes / **N**=No	
Who performs the VILIs?<br>1. Trained nurse<br>2. Midwife<br>3. Gynecologist<br>4. Others; please specify	

**Cervical Cancer Procedures**			
**Does your center provide the following services:**	**Y=Yes / N=No**	**Always available (>80% of the time) Indicate with =1**	**Not always available Indicate with =0**
Colposcopy			
Cervical biopsy			
Loop Electrical Excision Procedure (LEEP)			

Cryotherapy			
Cold knife cones			
HPV vaccine			
Others, please specify: (eg Cinluma)			

**Procedures**	**Y=Yes / N =No**	**Always available (>80% of the time) Indicate with =1**	**Not always available (indicate with =0**
Biopsy<br>1) Fine Needle Aspiration Cytology (FNAC)<br>2) Core Needle Biopsy/ Punch Biopsy<br>3) Excisional (surgical) Biopsy	1)<br>2)<br>3)	1)<br>2)<br>3)	1)<br>2)<br>3)
Does your facility conduct in-house pathology review?			
Does your facility conduct external pathology review?			
If external review, list facility name and country (if outside Gambia)			
Time for results?<br>1 <1 month<br>2 1-2 months<br>3 > 2 months			
Does your facility stain for immunohistochemistry?<br>1) ER	1)	1)	1)

2) PR<br>3) HER2	2)<br>3)	2)<br>3)	2)<br>3)
Does your facility perform breast/cervical cancer staging?			
Method of staging:<br>1 Clinical exam only<br>2 Clinical exam + Imaging<br>3 Pathological	 1)<br>2)<br>3)	 1)<br>2)<br>3)	 1)<br>2)<br>3)
Types of imaging used for staging<br>1) X ray<br>2) US-Scan<br>3) CT-Scan<br>4) MRI<br>5) PET-Scan	 1)<br>2)<br>3)<br>4)<br>5)	 1)<br>2)<br>3)<br>4)<br>5)	 1)<br>2)<br>3)<br>4)<br>5)
Does your facility perform breast cancer surgery?			
If yes:<br>1) Wide local excision/lumpectomy?<br>2) Mastectomy?<br>3) Axillary surgery?	 1)<br>2)<br>3)	 1)<br>2)<br>3)	 1)<br>2)<br>3)
If yes axillary surgery, Sentinel Lymph Node (SLN) mapping?			
If Yes SLN mapping, how?<br>1) Dye (write which one in comments if known)<br>2) Radio isotope (write which one in comments if known)	 1)<br>2)	 1)<br>2)	 1)<br>2)
Comments if any			

Does your facility perform surgery for cervical cancer			
If yes, which surgeries?<br>1) Simple hysterectomy<br>2) Radical hysterectomy<br>3) Trachelectomy	1)<br>2)<br>3)	1)<br>2)<br>3)	1)<br>2)<br>3)
**Reconstruction**			
Does your facility perform breast cancer reconstructive surgery?			
If yes, type of reconstructive surgery:<br>1) Expander/Implant<br>2) Rotational flap<br>3) Free flap	1)<br>2)<br>3)	1)<br>2)<br>3)	1)<br>2)<br>3)

**Oncology**			
	**Y=Yes / N=No**	**Always available (>80% of the time) Indicate with =1**	**Not always available (indicate with =0**
Does your facility offer chemotherapy?			
1. Chemotherapy for:<br>i Breast cancer only<br>ii Cervical cancer only<br>iii Both	 1)<br>2)<br>3)	 1)<br>2)<br>3)	 1)<br>2)<br>3)
If yes, which agents?<br>1 Cyclophosphamide<br>2 Doxorubicin (Adriamycin)<br>3 5FU<br>4 Methotrexate<br>5 Epirubicin<br>6 Docetaxel<br>7 Paclitaxel<br>8 Carboplatin<br>9 Cisplatin<br>10 Other, please specify	 1)<br>2)<br>3)<br>4)<br>5)<br>6)<br>7)<br>8)<br>9)<br>10)	 1)<br>2)<br>3)<br>4)<br>5)<br>6)<br>7)<br>8)<br>9)<br>10)	 1)<br>2)<br>3)<br>4)<br>5)<br>6)<br>7)<br>8)<br>9)<br>10)
Please indicate combinations used (such as CAF)			

Does your facility offer hormonal/ endocrine therapy?			
If yes, which agents?<br>1 Tamoxifen<br>2 Anastrozole<br>3 Esemestane<br>4 Fulvestrant<br>5 Goserelin<br>6 Letrozole<br>7 Trastuzumab(Herceptin)<br>8 Others (specify)	 1)<br>2)<br>3)<br>4)<br>5)<br>6)<br>7)<br>8)	 1)<br>2)<br>3)<br>4)<br>5)<br>6)<br>7)<br>8)	 1)<br>2)<br>3)<br>4)<br>5)<br>6)<br>7)<br>8)
Does your facility offer radiation therapy?			
If yes:<br>1 External beam<br>2 Brachytherapy<br>3 Others; please state	 1)<br>2)<br>3)	 1)<br>2)<br>3)	 1)<br>2)<br>3)

**Miscellaneous**	**Y=Yes / N=No**	1=Ready available	0=Not readily available
Does your facility offer long term follow up?			
Does your facility have a registry of cancer patients?			
Does your facility conduct cancer outreach?			
Does your facility offer counseling services?			
Does your facility offer palliative care services?			
